# Inhibitory effect of *Isatis tinctoria L.* water extract on DNCB-induced atopic dermatitis in BALB/c mice and HaCaT cells

**DOI:** 10.1186/s13020-022-00624-5

**Published:** 2022-06-08

**Authors:** Ga-Yul Min, Tae In Kim, Ji-Hye Kim, Won-Kyung Cho, Ju-Hye Yang, Jin-Yeul Ma

**Affiliations:** Korean Medicine (KM) Application Center, Korea Institute of Oriental Medicine, 70 Cheomdan-ro, Dong-gu, Daegu, 41062 Republic of Korea

**Keywords:** *Isatis tinctoria L*, Atopic dermatitis, BALB/c mice, HaCaT cells

## Abstract

**Background:**

*Isatis tinctoria L* (PLG) is a medicinal herb from the roots of *Isatis indigotica Fort* (Family Cruciferae). Previous studies have shown that PLG has anti-inflammatory and therapeutic effects against conditions such as acute and chronic hepatitis, various respiratory inflammations, and cancer. The purpose of this study was to define the pharmacological effects of PLG on inflammatory reactions and skin hyperkeratosis, which are the main symptoms of atopic dermatitis (AD), in vivo and in vitro.

**Methods:**

For the AD in vivo experiment, 2,4-dinitrochlorobenzene (DNCB) induction and oral administration of PLG were performed on male BALB/c mice for four weeks. For in vitro experiments, keratinocytes were activated using TNF-α/IFN-γ in cultured human keratinocyte (HaCaT) cells. PLG inhibited inflammatory chemokine production and blocked the nuclear translocation of NF-κB p65 in activated keratinocytes.

**Results:**

As a result of oral administration of PLG, dermis and epidermis thickening, as well as eosinophil and mast cell infiltration, were attenuated in AD skin lesions. In addition, the levels of immunoglobulin E (IgE), pro-inflammatory cytokines, and the MAPK/NF-κB signaling pathway were decreased in serum and dorsal skin tissues. Furthermore, PLG inhibited inflammatory chemokine production and blocked the nuclear translocation of NF-κB p65 in activated keratinocytes. In addition, epigoitrin and adenosine, the standard compounds of PLG, were identified as candidate AD compounds.

**Conclusions:**

These results indicate that PLG is a potent therapeutic agent for attenuating symptoms of AD.

**Supplementary Information:**

The online version contains supplementary material available at 10.1186/s13020-022-00624-5.

## Background

Atopic dermatitis (AD) is a chronic recurrent inflammatory skin disease caused by an imbalance in the immune response due to genetic and environmental factors [1, 2]. AD is increasing in prevalence worldwide, affecting 2%–4% of adults and up to 15%–20% of children [3]. Chronic dermatitis is characterized by severe symptoms such as erythema, itchiness, dryness, and skin hypersensitivity, and can lead to mental distress, sleep disturbance, and reduced quality of life [4–6].

Studies have shown that the immunological mechanism of AD is skin inflammation caused by T cell activation [1]. In AD, Th2 cells are largely predominant in the acute phase, and then converted to Th1 cells in the chronic phase [7]. In the early stages of the disease, T cells involved in immune response regulation secrete pro-inflammatory cytokines (such as IL-4, IL-5, and IL-13), causing naïve CD4 + T cells to differentiate into Th2 cells [8, 9]. Cytokines secreted by Th2 cells promote activation of eosinophils and B cells, and increase the production of IgE [10]. IgE binds to the IgE receptor attached to the surface of mast cells, and activates mast cells to release various pro-inflammatory cytokines, histamines, and chemokines [11–13]. Keratinocytes are cells that play an important role in skin diseases, and they stimulate epidermal keratinocytes with pro-inflammatory cytokines such as TNF-α/IFN-γ to produce inflammatory chemokines [14]. In addition, activation of inflammatory signaling pathways such as MAPK/NF-κB induce the synthesis of inflammatory chemokines, leading to skin inflammation [15]. Therefore, downregulation of chemokines through inhibition of inflammatory signaling pathways may be an important strategy in the treatment of inflammatory skin diseases [16, 17].

The most widely used AD treatments to date include corticosteroids, antihistamines, and immunosuppressive drugs [18]. However, continuous administration of these drugs may cause various side effects, such as skin atrophy, osteoporosis, and bacterial/viral skin infections [19]. Therefore, there is still a need to develop new drugs that are safe and effective alternatives to these options.

*Isatis tinctoria L* (PLG), the root of Isatis indigotica Fort, called “Panlamgeun,” has been used in traditional Chinese medicine for thousands of years to treat acute and chronic infections, various respiratory infections, and cancer [20, 21]. PLG contains many compounds such as indigo, indigotin, indirubin, Epigoitrin, Adenosine, and L-Arginine [21–24]. In particular, indigo and indirubin have been reported to have anti-tumor, anti-inflammatory, and immune-enhancing effects of polysaccharides [25, 26]. Further, Epigoitrin has been used as a therapeutic marker for antivirals in Chinese Pharmacopoeia, and adenosine is known to be a major nucleoside of PLG [21, 27, 28]. Recently, nucleosides have been shown to be bioactive compounds associated with immunomodulatory, antiviral, and anti-inflammatory diseases [29–31]. In a previous study, PLG showed a pharmacological effect of inhibiting the activity of iNOS and COX-2, by suppressing the expression of the MAPK/NF-κB pathway in LPS-induced Raw264.7 cells [20]. PLG is known to be effective against inflammation, but the effect on anti-atopic skin inflammation has not yet been confirmed. And so, we investigated the effect of PLG on AD in DNCB-induced BALB/c mice and TNF-α/IFN-γ-stimulated HaCaT cells, and elucidated the immunological mechanism of action. We also performed HPLC analysis to search for active compounds and their contents in PLG.

## Materials and methods

### Materials

*Isatis tinctoria L* (PLG) was obtained from the Yeongcheon Oriental Herbal Market (Yeongcheon, Korea). DNCB, protease inhibitor cocktail and phosphatase inhibitor cocktail were purchased from Sigma‑Aldrich (St. Louis, MO, USA). Standard compound, Epigoitrin (CFN 99521, purity ≥ 98%) was purchased from chemfaces chemfaces (Wuhan, Hubei, China) and Adenosine (A9251, purity ≥ 99%) was obtained from sigma Aldrich (St. Louis, MO, USA). Methanol solvent used in HPLC analysis was supplied from JT Baker (Philiosburg, NJ, USA). Distilled Water was prepared a Puris-Evo RO ultrapure water system (Mirae ST Co., Ltd., Anyang-si, South Korea). Mouse IgE (cat. no. 555248), Mouse TNF (cat. no. 555268) ELISA kits were purchased from BD Biosciences (Franklin Lakes, NJ, USA). Antibodies to phosphorylation-ERK (p-ERK), ERK, phos-phorylation-P38 (p-P38), P38 and NF-κB were purchased from Cell Signaling (Danvers, MA, USA). Recombinant Human TNF-α/IFN-γ Protein was purchased from R&D Systems (Minneapolis, MN, USA). Human RNATES, MDC and TARC were obtained from Bio-Legend (San Diego, CA, USA). Primer and AccuPower CycleScript RT PreMix were purchased from Bioneer (Daejeon, Korea), MTT (EZ-Cytox) was purchased from Daeil Lab (Chung-cheong bukdo, Korea).

### Preparation of PLG water extract

*Isatis tinctoria L* (50 g) was extracted with hot water for 3 h with 1 L of mineral water. The extract was filtered through a stainless steel filter and then freeze-dried for seven days. The PLG powder was stored in a − 20 °C freezer before use.

### Animals and induction of AD-like lesions and drug treatment in mice

Male six-week-old BALB/c mice (18–20 g) were obtained from Samtako BioKorea (Osan, Korea). All animal experimental conditions were performed at a temperature of 22.5 °C ± 0.5 °C and a humidity of 42.6% ± 1.7%, maintaining a 12 h light–dark cycle, and food and water were provided ad libitum. All mouse procedures were performed with the approval of the Korea Institute of Oriental Medicine Animal Care and Use Committee (No. 21–044).

**Fig. 1:**
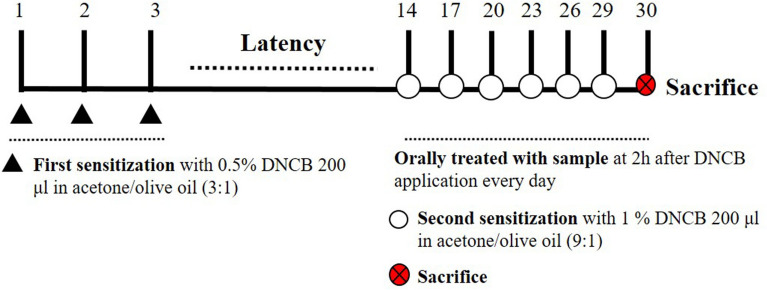
Experimental schedule (schematic)

The AD animal experiment was conducted for 30 days [32]. DNCB was applied to the dorsal skin, and the drug was orally administered. BALB/c mice were randomly divided into five groups: Normal (vehicle—treated), Control (DNCB—induced), PLG 100 (DNCB + PLG 100 mg/kg), PLG 200 (DNCB + PLG 200 mg/kg), Dexamethasone (DNCB + DEX 1 mg/kg). During the first sensitization, phosphate-buffered saline was applied to the dorsal skin in the Normal group, and the Control, PLG 100, 200 mg/kg, and DEX groups were sensitized to 0.5% DNCB three times consecutively on the dorsal skin. During the secondary sensitization, 0.5% Carboxymethyl cellulose (CMC) was orally administered to the Normal group, and the Control, PLG 100, 200 mg/kg, and DEX groups were sensitized to 1% DNCB once every three days, and oral administration of the drug was performed for 16 days **(**Fig. 1**)**. On day 30, mice were euthanized by intraperitoneally injecting 400–500 μl (based on 25 g of a mouse, proceeding at a concentration of 250 mg/kg) by diluting avertin (2, 2, 2-Tribromoethanol) in 2-methyl-2-butanol. Blood samples were collected from the heart, coagulated at room temperature for one hour, and then centrifuged at 2000 rpm–4 °C–10 min to separate serum. Dorsal skin tissues were collected for genetic analysis and histological examination.

### Histological analysis

For histological evaluation, dorsal skin tissues were fixed in 10% Neutral Buffered Formalin for one day. The fixed tissue was commissioned to a commercial company (Garam Meditech, Korea) for hematoxylin and eosin (H&E) and toluidine blue (TB) staining. Then, using a microscope (Nikon Eclipse Ti; Nikon, Tokyo, Japan), epidermal and dermal thickness, as well as eosinophil and mast cell infiltration were observed. The thickness of the dermis and epidermis was analyzed (H&E; magnification,× 100; 3 fields per section). Eosinophils (H&E; magnification, × 400, 6 fields per section) and mast cells (TB; magnification, × 200, 3 fields per section) distributed in the dermis were observed. The skin thickness and the number of inflammatory cells were measured using ImageJ software (version 1.46; National Institutes of Health).

### Measurement of serum IgE and TNF-α levels

The serum collected on the day of euthanasia was stored at − 80 °C. IgE and TNF-α levels were measured using the BD bioscience ELISA kit (Franklin Lakes, NJ, USA). All experiments and results were performed according to the manufacturer’s instructions.

### Western blot

Total-protein was extracted from frozen mouse skin tissues using T-PER™ Tissue Protein Extraction Reagent and Extraction Buffer (cat. no. 78510, Thermo Fisher Scientific Inc). In addition, lysis was performed using NE-PER™ Nuclear and Cytoplasmic Extraction Reagents (cat. no. 78835, Thermo Fisher Scientific Inc) for nuclear separation. The extracted protein (30 µg) was separated by gel electrophoresis (10% gel) and transferred to Protran nitrocellulose (NC) membranes. The membrane was blocked using 5% skim milk and 3% BCA, and incubated with the primary antibody at 4 °C. Protein expression was detected with the ChemiDoc Imaging System (Bio-Rad) using a chemiluminescent detection reagent for specific protein detection. Antibody information is shown in Table 1.

**Table 1:** Primary antibody list for Western blot analysis

Antigen	Supplier	Working dilution	Cat. No
p-ERK	Cell signalling	1:1000	4370
t-ERK	Cell signalling	1:1000	9102
p-p38	Cell signalling	1:1000	9211
t-p38	Cell signalling	1:1000	9212
β-Actin	Santa Cruz	1:1000	Sc-517582
NF-κB p65	Cell signalling	1:1000	8242
Lamin B1	Cell signalling	1:1000	12586
HRP- conjugated IgG	Invitrogen	1:10000	31460

### Cell culture

HaCaT cells were maintained in a culture medium diluted with 10% fetal bovine serum and 1% Penicillin–Streptomycin (p/s) in Dulbecco’s modified Eagle medium at 37 °C in 5% CO_2_.

### Cell viability assay

Sample preparation for in vitro experiments was as follows. PLG powder was dissolved in distilled water at room temperature for two hours and centrifuged at 13,000 rpm–4 °C–10 min. The resulting supernatant was transferred to a clean 1.5 ml EP tube, and stored at− 20 °C until use. HaCaT cells were seeded in a 96 well plate, with 8 × 10^3^ cells/well. After 24 h, PLG (100, 200, and 400 μg/mL) or Adenosine (10, 30, and 50 μM) and Epigoitrin (100, 200, and 400 μM) was added to the medium for 24 h. Then, 20 μL of the EZ-Cytox solution (Daeil Lab; Chung-cheong bukdo, Korea) were added to each well. After incubation for two hours at 37 °C, the optical density was measured using an ELISA (Infinite M200, Tecan, Männedorf, Switzerland) at an optical density of 450 nm.

### Detection of secretory chemokines

HaCaT cells were seeded at 8 × 10^4^ cells/well in a 24-well plate and incubated overnight. Thereafter, PLG (100, 200, and 400 μg/mL) or Adenosine (10, 30, and 50 μM) and Epigoitrin (100, 200, and 400 μM) was pretreated for 1 h, and TNF-α/IFN-γ (10 ng/mL) was stimulated for 24 h. To collect the supernatant and remove particulates, the cell culture medium was centrifuged for 12,000 rpm–4 °C–10 min. The levels of RANTES/CCL5 and TARC/CCL17 in the cell culture supernatant were detected using a Bio-Legend ELISA kit (San Diego, CA, USA), and MDC/CCL22 and MIP-3α/CCL20 were measured using an R&D Systems ELISA kit (Minneapolis, MN, USA). In addition, the protein concentration of MCP-1/CCL2 was analyzed using the BD bioscience ELISA kit (Franklin Lakes, NJ, USA). All results were performed according to the manufacturer’s instructions, and standard curves were performed using standard samples from the kit.

### Real-time reverse transcription PCR (RT-PCR) analysi*s*

Total RNA was extracted from HaCaT cells and dorsal skin tissue. HaCaT cells were seeded at 4 × 10^5^ cells/well in a six-well plate and incubated overnight. PLG (100, 200, and 400 μg/mL) was then pretreated for 1 h, and TNF-α/IFN-γ (10 ng/mL) was stimulated for 24 h. RNA was extracted from the dorsal skin tissues and cells using the easy-BLUE™ Total RNA Extraction Kit (Intronbio; Gyeonggi-do, Korea) and synthesized into cDNA using AccuPower CycleScript RT PreMix (Bioneer; Daejeon, Korea). Real-time PCR was performed with primer and AccuPower® Pfu PCR premix (Bioneer). The PCR conditions were as follows: Initial denaturation at 95 °C for 5 s, primer annealing followed by 40 cycles at 62.5 °C for 30 s. Relative gene expression levels of mRNA were calculated using the ΔΔCt method. Primer sequences are listed in Table 2.

**Table 2:** Primer sets for real-time RT-PCR

Name	Forward	Reverse
h-RANTES	5′ GATGCCAAAG AGAGAGGGAC 3′	5′ AAATTTGTGT AAGTTCAGGT 3′
h-TARC	5′ CTGCACACAG AGACTCCCTC 3′	5′ CTGGTACCAC GTCTTCAGCT 3′
h-MDC	5′ GAAACACTTC TACTGGACCT 3′	5′ CAGGGAGGTA GGGCTCCTGA '
h-GAPDH	5′ TCAAGGCTGA GAACGGGAAG 3′	5′ TGGACTCCAC GACGTACTCA 3′
m-TNF-α	5′ ATGAGCACAG AAAGCATGAT 3′	5′ TACAGGCTTG TCACTCGAAT 3′
m-IL-6	5′ TTCCATCCAG TTGCCTTCTT 3′	5′ ATTTCCACGA TTTCCCAGAG 3′
m-IL-13	5′ CATCTCCAAT TGCAATGCCA ′	5′ GCCCAGGGAT GGTCTCTCCT 3′
m-GAPDH	5′ AACGACCCCT TCATTGAC 3′	5′ TCCACGACAT ACTCAGCAC 3′

*h* human, m mouse, *RANTES* regulated on activation normal T cell expressed and secreted, CCL5 TARC, thymus and activation-regulated chemokine, *CCL17*, *MDC* macrophage-derived chemokine, *CCL22*

### Immunocytochemistry (ICC) analysis

Cell seeding and fixation conditions were based on previous studies [33]. The fixed cells were treated overnight with NF-κB p65 primary antibodies (Cell Signaling; MA, USA). Afterwards, cells were reacted with secondary Alexa Fluor™ 488 Antibodies for 20 min (cat no. A11001; Thermo Fisher Scientific Inc; MA, USA). Finally, cells were stained with DAPI solution for 10 min at room temperature (40,6-diamidino-2-phenylindole, cat no. 8417; Sigma-Aldrich Inc; MA, USA). All samples were then observed using a confocal microscope (FV3000 FLUOVIEW, Olympus, Tokyo, Japan).

### Preparation of standard and samples

An epigoitrin and adenosine standard solution was prepared at a concentration 1000 ppm using methanol, and diluted with a solvent to prepare a standard curve for each concentration. The PLG extract was dissolved with water (HPLC grade) for 30 min using an ultrasonicator (JAC Ultrasonic JAC-3010) at 100 mg/ml concentration. After extraction, PLC was filtered through a 0.2 PVDF membrane. Aliquot of filtrate was injected for HPLC analysis.

All HPLC analysis was conducted with the Dionex Ultimate 3000 system and assembled with a department of column oven, a binary pump, an auto sampler, and a DAD-UV detector (Dionex Corp., Sunnyvale, CA, USA). HPLC chromatogram data was processed using Chromeleon 7 software (Thermo Fisher, Counteaboeuf, France).

To confirm the contents of epigoitrin and adenosine in PLG extract, an HPLC analysis was performed. The chromatography column was an X-bridge C18 column (250 mm × 4.6 mm, 5 μm) equipped with a C18 guard cartridge (4.0 mm ×3.0 mm). Mobile phase, A: water B: methanol eluted with gradient method: 0–12 min, 3%–10% B; 12–17 min, 10%–20% B; 17–25 min 20% B; 25–35 min, 20%–30% B; 35–40 min 30%–40% B; 40–50 min 40%–100% B. Flow rate was 1 ml/min, column temperature was 25 °C, injection volume was 10 μl, and UV detection was 245 nm (Additional file 1: Table S1). Identification of the standard compounds was confirmed using retention time and UV spectra under the same HPLC analysis conditions. HPLC conditions for analytical standard compounds and PLG extracts are specified in Additional file 1: Table 1.

### Statistical analysis

All experiments were repeated at least three times. Statistical analysis was performed us-ing GraphPad Prism Software (version 5.01; GraphPad Software, Inc.). Data are presented as mean ± SEM assessed using Student's t-test or analysis of variance (one-way ANOVA, Dunnett, control comparison). P < 0.05 was considered to indicate a statistically significant difference.

## Results

### Effects of PLG on symptoms in DNCB-induced mice

**Fig. 2:**
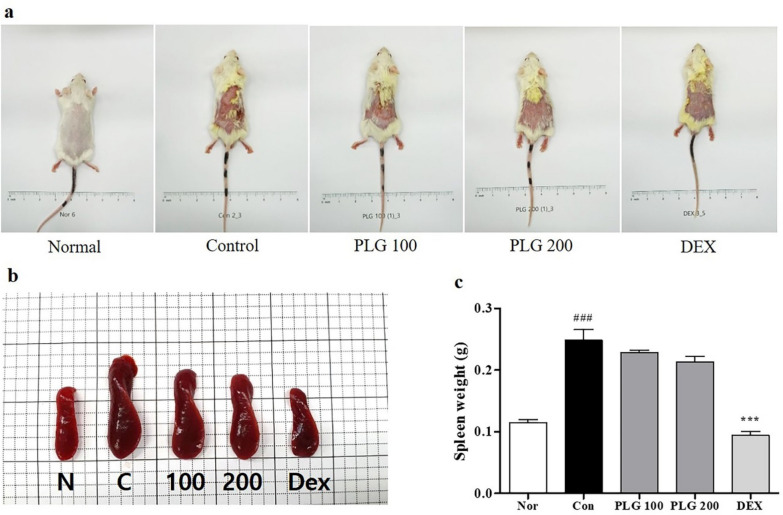
Effects of PLG on dorsal skin lesions and spleen weight in DNCB-induced mice. **a**, **b** Dorsal skin lesions and spleens images for each treatment group on day 30. **c** Measurement of spleen weight. Data represent the mean ± SEM. *n* = 10. ^###^*P* < 0.001 vs. Normal group; ^***^P < 0.001 vs. DNCB-induced group

Through images of dorsal skin lesions, it was visually confirmed whether PLG affected the symptoms of AD in DNCB-induced BALB/c mice (Fig. 2a). In the Control group, the symptoms of erythema, edema, erosion, and dryness of the skin lesions worsened compared to the Normal group, and the symptoms in the PLG treatment group were improved compared to the Control group. One of the hallmarks of chronic inflammatory skin disease is hypertrophy of immune organs such as the spleen [34]. Spleen size and weight were measured to confirm immune system status (Fig. 2b, c). The weight and size of the spleen isolated from DNCB mice were significantly enlarged compared to the Normal group. Moreover, the spleen hypertrophy was significantly reduced in the PLG-treated group.

### Effects of PLG on epidermis and dermis thickness in DNCB-induced mice

**Fig. 3:**
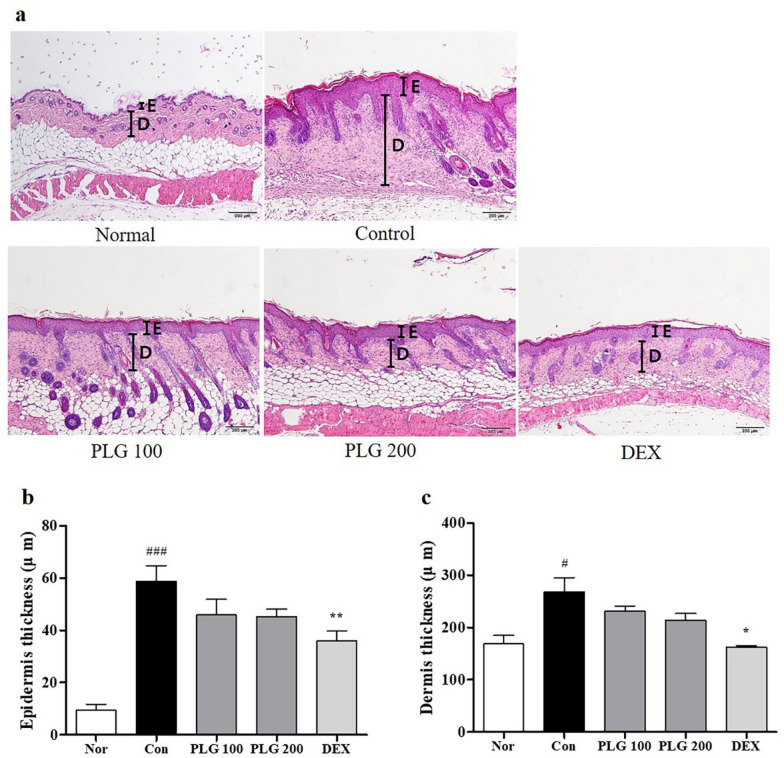
Effects of PLG on epidermis and dermis thickness in DNCB-induced mice. **a** Epidermis and dermis thickness was examined by H&E staining (Magnification, × 100. Scale bar, 200 µm). **b**, **c** Measurement of epidermal and dermal thickness. Data represent the mean ± SEM. *n* = 10. ^#^*P* < 0.05 and ^###^*P* < 0.001 vs. Normal group; ^*^P < 0.05 and ^**^P < 0.01 vs. DNCB-induced group

To determine whether PLG reduces the thickness of the epidermis and dermis in AD-induced skin lesions, H&E staining was performed on the dorsal skin tissue (Fig. 3a). In the DNCB-induced Control group, the thickness of the epidermis and dermis was significantly increased compared to the Normal group, and it was observed that the thickness of the epidermis and dermis was significantly decreased in the PLG-treated group (Fig. 3b, c).

### Effects of PLG on immune cell infiltration in DNCB-induced mice

**Fig. 4:**
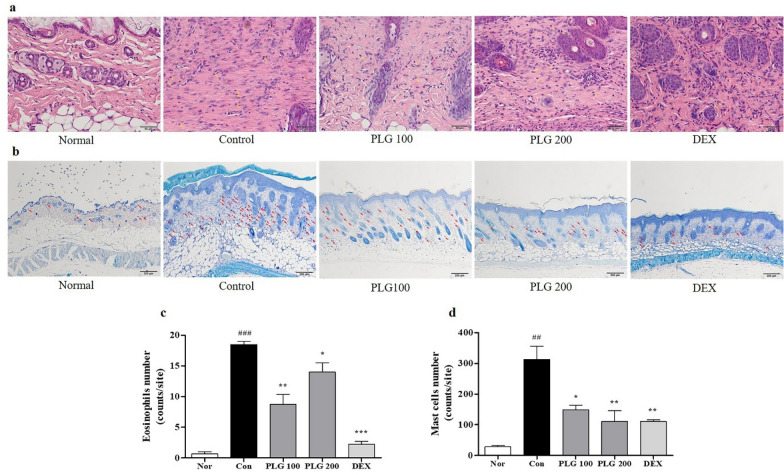
Effects of PLG on immune cell infiltration in DNCB-induced mice. **a**, **b** Infiltration of eosinophils and mast cells into dermis lesions was examined by H&E and TB staining (H&E; Magnification, × 400; scale bar, 50 µm) (TB; magnification,× 100; scale bar, 200 µm). **c**, **d** The number of immune cells was counted using ImageJ software. Data represent the mean ± SEM. *n* = 10. ^##^*P* < 0.001 and ^###^*P* < 0.01 vs. Normal group; ^*^P < 0.05, ^**^P < 0.01 and ^***^P < 0.001 vs. DNCB-induced group

H&E and TB were performed to confirm the infiltration effect of mast cells and eosinophils in the dermis (Fig. 4a, b). Eosinophil infiltration was increased in the Control group compared to the Normal group. Moreover, the PLG treatment group significantly reduced the infiltration of eosinophils (Fig. 4c). Mast cell infiltration was increased in the Control group compared to the Normal group. In contrast, the PLG treatment group significantly reduced the infiltration of mast cells (Fig. 4d).

### Effects of PLG on serum IgE and pro-inflammatory cytokines in DNCB-induced mice

**Fig. 5:**
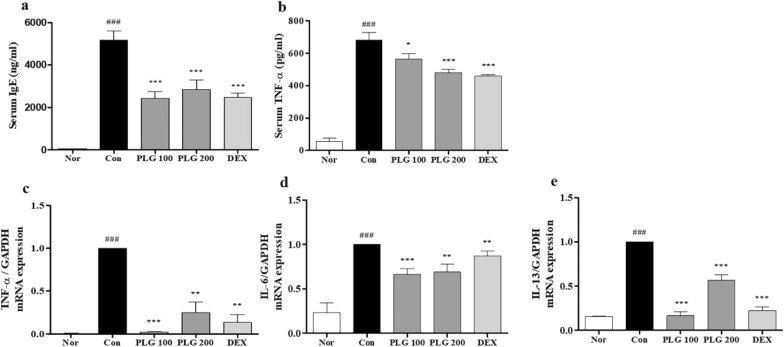
Effects of PLG on IgE and pro-inflammatory cytokines in DNCB-induced mice. **a**, **b** Serum levels of IgE and TNF-α were quantified by ELISA. **c**–**e** Production of TNF-α, IL-6, and IL-13 was determined by real-time RT-PCR. Data represent the mean ± SEM. *n* = 10. ^###^*P* < 0.01 vs. Normal group; ^*^P < 0.05, ^**^P < 0.01 and ^***^P < 0.001 vs. DNCB-induced group

ELSIA and real-time RT-PCR were performed to measure the expression of IgE and TNF-α in serum and pro-inflammatory cytokines in dorsal tissues (Fig. 5). The expression of IgE and TNF-α in the serum was significantly increased in the Control group compared with the Normal group, and was significantly decreased in the PLG-treated group (Fig. 5a, b). As a result of measuring the mRNA expression of pro-inflammatory cytokines (such as TNF-α, IL-6, IL-13) in the skin tissue, the Control group was significantly increased compared to the Normal group, and the PLG-treated group was significantly decreased (Fig. 5c–d).

### Effect of PLG on MAPK/NF-κB expression in DNCB-induced mice

**Fig. 6:**
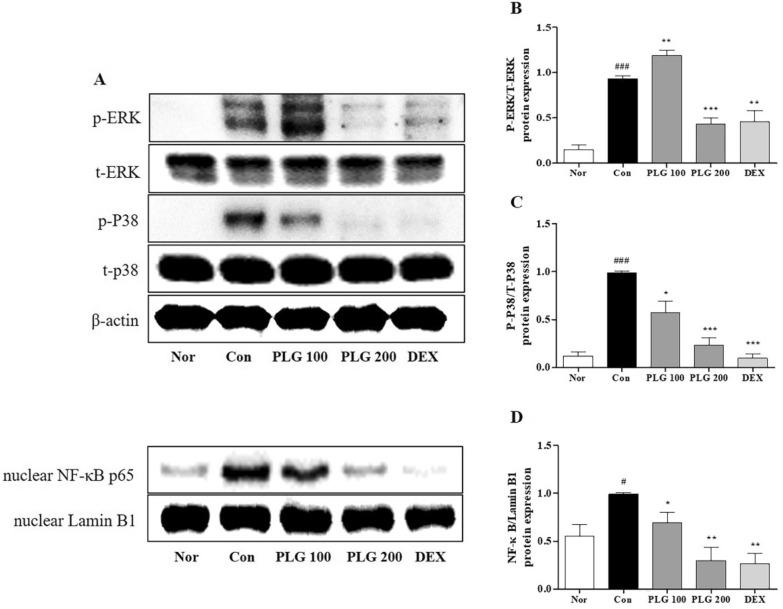
Effect of PLG on MAPK/NF-κB expression in TNF-α/IFN-γ-induced HaCaT cells. **a** p-EKR, p-P38 and NF-κB signaling pathways were determined by western blot. **b**-**d** Evaluation of MAPK/NF-κB expression level by image J. Data represent the mean ± SEM. ^###^*P* < 0.05 and ^###^*P* < 0.01 vs. Normal group; ^*^P < 0.05, ^**^P < 0.01 and ^***^P < 0.001 vs. Control group

To confirm the anti-inflammatory modulating effects of PLG, protein was extracted from the dorsal tissue and the expression of MAPK/NF-κB signaling pathways was observed (Fig. 6a). In AD-induced skin tissue, phosphorylation of extracellular signal-regulated kinase (ERK) and p38 was significantly increased. Further, in the PLG-treated group, ERK decreased only at high concentrations and p38 decreased in a concentration-dependent manner (Fig. 6b, c). Furthermore, the expression of NF-κB p65 in the AD-induced skin tissue was significantly increased, and decreased significantly in the PLG-treated group (Fig. 6d).

### Effect of PLG on chemokine production in HaCaT keratinocytes

**Fig. 7:**
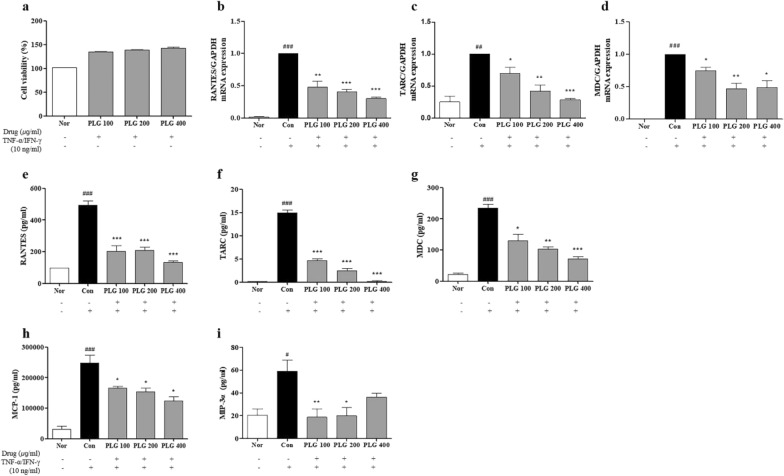
Effects of PLG on chemokine production in TNF-α/IFN-γ-induced HaCaT cells. **a** The cytotoxicity of PLG was determined by MTT assay. **b**–**d** Expression of RNATES, TARC, and MDC was analyzed with real-time RT-PCR. **e**–**i** Production of RNATES, TARC, MDC, MCP-1, and MIP-3α was examined by ELISA. Data represent the mean ± SEM of three independent experiments. ^#^*P* < 0.05 ^##^*P* < 0.01 and ^###^*P* < 0.001 vs. Normal group; ^*^P < 0.05, ^**^P < 0.01 and ^***^P < 0.001 vs. Control group

After confirming the pharmacological efficacy of PLG in AD mice, it was further confirmed that PLG affects the molecular mechanism using HaCaT cells (Fig. 7). Before the experiment, various concentrations of PLG (100, 200, and 400 μg/mL) were treated in HaCaT cells for 24 h to check the cytotoxicity by PLG. As confirmed by EZ-Cytox analysis, it showed no cytotoxicity at high concentrations of up to 200 μg/mL (Fig. 7a). Real-time RT-PCR and ELISA were performed to confirm the effect of PLG on chemokines related to AD. As a result of PCR, the mRNA expression of RANTES, TARC, and MDC was significantly increased in the Control group compared to the Normal group. In contrast, gene expression was significantly reduced in the PLG-treated group (Fig. 7b–d). Concentrations of RANTES, TARC, MDC, MCP-1, and MIP-3α in the culture medium were significantly increased in the Control group compared to the Normal group, and significantly decreased in the PLG-treated group (Fig. 7e–i).

### Effects of PLG on NF-κB p65 translocation in HaCaT keratinocytes

**Fig. 8:**
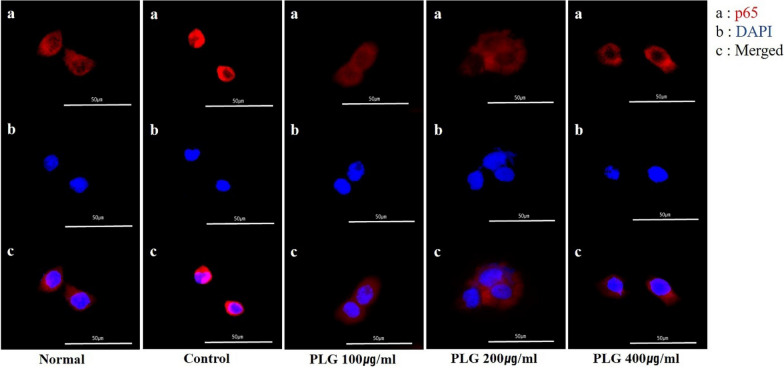
Effects of PLG on NF-κB p65 translocation in TNF-α/IFN-γ-induced HaCaT cells. **a**–**c** Translocation of p65 was determined using a fluorescent microscopy. Representative photomicrographs of NF-κB p65 (red, a), DAPI (blue, b) and Merged imaged (merged, c) in the TNF-α/IFN-γ-induced HaCaT cells

Immunocytochemical staining was performed to confirm the nuclear translocation of NF-κB p65, a transcription factor critically involved in signal transduction in AD. As a result, the expression of NF-κB p65 in the nucleus was increased in HaCaT cells stimulated with TNF-α/IFN-γ. Moreover, it was confirmed that the PLG-treated group inhibited the expression of NF-κB p65 in the nucleus, blocking nuclear translocation (Fig. 8).

### High-performance liquid chromatography analysis

**Fig. 9:**
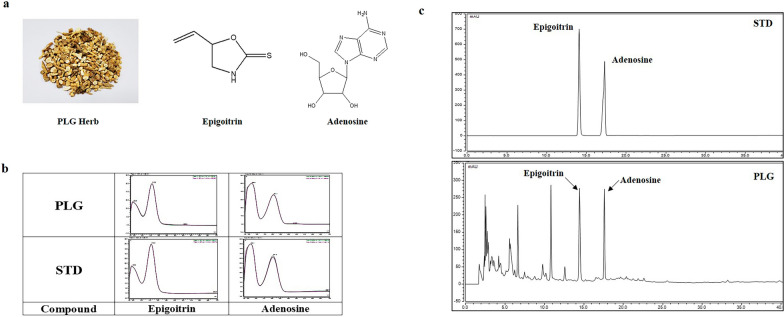
**a** Chemical structure of Epigoitrin and Adenosine, PLG herbal medicine, (**b**) UV spectra of PLG extract and standard compounds at 245 nm, (**c**) HPLC chromatogram of PLG extract and standard mixture

Two standard compounds were detected on developed HPLC conditions 14.367 and 17.533 min (Fig. 9), and were detected in the PLG extract as well. Based on these results, we found that epigoitrin and adenosine were present in PLG extract. The calibration curves of the two compounds were y = 1.2153x + 0.4749, and y = 0.3532x + 0.1549 with coefficients of determination 0.9997 and 1.0000 at injected concentration ranges of 10.0–100.0 μg/mL (Epigoitrin) and 20.0–200.0 μg/mL (Adenosine), respectively (Table 3). As a result, the calibration curve of the compounds had good linearity at the concentration ranges. The contents of two constituents were confirmed, and the peak area mean value in the PLG extract was calculated using a calibration curve prepared for each concentration. In these results, the concentrations of epigoitrin and adenosine were 0.037% and 0.085%, respectively. According to Chinese Pharmacopoeia, 2020 edition, epigoitrin is a marker compound of Isatidis Radix, and it was detected in our PLG extract as well.

**Table 3:** Calibration curves of constituents

Compound	Range.(ug/mL,ppm)	Regression equation	*r*^2^	LOD(μg/ml)	LOQ(μg/ml)
1	10.0 ~ 100.0	y = 1.2153x + 0.4749	0.9997	0.0091	0.0276
2	20.0 ~ 200.0	y = 0.3532x + 0.1549	1.0000	0.0313	0.0950

Epigoitrin (**1**); Adenosine (**2**)

LOD = 3.3 × σ/ *S*. LOQ = 10 × σ / *S*. σis the standard deviation of the intercept from the regression equation and *S* is the slope of the calibration curve

### Effect of adenosine and epigoitrin on chemokine production in HaCaT keratinocytes

**Fig. 10:**
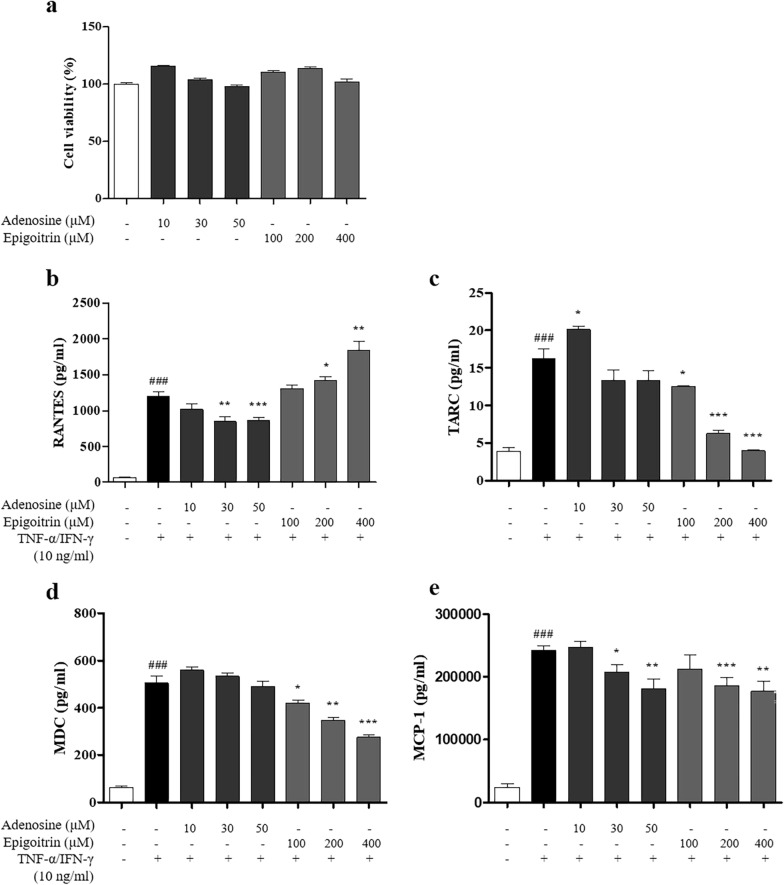
Effects of PLG standard compound Adenosine and Epigoitrin on the production of pro-inflammatory chemokine in TNF-α/IFN-γ-induced HaCaT cells. **a** The cytotoxicity of Adenosine and Epigoitrin was determined by MTT assay, **b**–**e** Production of RANTES, TARC, MDC and MCP-1 was examined by ELISA. Data represent the mean ± SEM of three independent experiments. ^###^*P* < 0.001 vs. Normal group; ^*^P < 0.05, ^**^P < 0.01 and ^***^P < 0.001 vs. Control group

To determine the active compounds of PLG, we investigated the effects of adenosine and epigoitrin on inflammatory chemokine production in TNF-α/IFN-γ-induced HaCaT cells (Fig. 10). In order to confirm the cytotoxicity of the two compounds before the experiment, cell viability analysis was performed by treatment for 24 h (Fig. 10a). Based on these results, the concentrations of adenosine (10, 30, and 50 μM) and epigoitrin (100, 200, and 400 μM) were determined and the experiment was carried out. ELISA was performed to investigate the effects of adenosine and epigoitrin on AD-associated inflammatory chemokine production (Fig. 10b-e). The concentrations of RANTES, TARC, MDC and MCP-1 in the culture medium were significantly increased in the Control group compared to the Normal group. Adenosine compound showed the effect of reducing the concentration of RANTES, TARC, and MCP-1, and the epigoitrin compound confirmed the effect of inhibiting the concentration of TARC, MDC and MCP-1.

## Discussion

The prevalence of AD has been increasing for decades, leading to complications, anxiety, and poor quality of care [35]. Successful treatment for AD requires a variety of approaches, including reducing immune imbalance and treating the inflammation and itchiness [36]. Corticosteroids and calcineurin inhibitors, which are currently established AD treatment agents, immediately relieve symptoms, but can cause side effects with long-term use [37, 38]. Therefore, it is essential to use a therapeutic agent with reduced side effects and increased safety. PLG, a natural product, is known to have anti-inflammatory effects. Previous research has hypothesized that PLG has potential for the treatment of inflammatory skin disease and immune modulation. Therefore, the purpose of this study is to investigate whether PLG is effective in treating AD in DNCB-induced atopic dermatitis-like animal models and TNF-α/IFN-γ-stimulated HaCaT cell models.

An in-depth study was conducted on the pathogenesis of atopic dermatitis in an animal model of DNCB-induced AD. BALB/c mice induced via DNCB are characterized by a Th2 immune response and are widely used in the field of immunology [39]. In addition, DNCB increases the expression of various cytokines and chemokines that cause AD [40, 41]. The skin lesions of chronic AD have many characteristics, such as dryness, epidermis and dermis thickness, and lichenification, and AD induces various responses within the immune system, affecting the weight of immune organs such as the spleen [42, 43]. We demonstrated that PLG reduced the severity of lesions on mice skin, epidermal and dermal thickening, and spleen weight, and demonstrated that PLG suppressed immune responses and improved histological characteristics.

The main pathogenesis of AD is the disruption of epidermal barrier function and excessive infiltration of allergens leading to inflammation, a hallmark of IgE-mediated hypersensitivity reactions [44]. Activation of T helper (Th) cells leads to cytokine production and inflammatory cell infiltration due to an imbalance between Th1 and Th2 cells [45, 46]. It is also known that the immune response of Th2 cells plays a pivotal role in AD [7]. Th2 cell cytokines (such as IL-6, IL-13) play an important role in the disease of AD and increase the production of IgE by activating B cells [47]. The generated IgE binds to the high-affinity IgE-Fc receptor type I (FcϵRI) on the mast cell surface and activates the mast cell [48]. Activated mast cells release inflammatory mediators such as histamines, chemokines, and pro-inflammatory cytokines [48]. Furthermore, eosinophil infiltration into skin lesions via Th2 cell cytokines is another important feature of AD [48]. In this study, PLG decreased the levels of IgE and TNF-α in serum and inhibited the mRNA expression of TNF-α, IL-6, and IL-13 in skin tissues. Furthermore, it improved the infiltration of mast cells and eosinophils into the dermis of the skin tissue. These experimental results indicate that PLG can inhibit the expression of IgE, pro-inflammatory cytokines, and infiltration of inflammatory cells (such as mast cells and eosinophils) by inhibiting the expression of Th2 cell cytokines.

MAPKs, including ERK, and p38, are implicated in the inflammatory signaling cascade and are essential for the pathogenesis of the inflammatory response [13, 49, 50]. Phosphorylation of MAPK is an important pathway in inhibiting inflammation because it promotes the inflammatory response by generating inflammatory mediators [13]. Moreover, signaling activation of MAPK plays an important role in NF-κB activation [51, 52]. NF-κB is a transcription factor known as a key indicator of the progression of inflammatory dermatitis. [53–56]. Therefore, the expression of NF-κB can be a measure of the treatment of inflammatory diseases as well as AD [57]. Under normal conditions, NF-κB p65 is bound to IκBα in the cytoplasm, and when stimulated by cytokines such as TNF-α/IFN-γ, it promotes the degradation and phosphorylation of IκBα, thereby increasing the phosphorylation and nuclear translocation of NF-κB p65 [58]. Nuclear translocated NF-κB p65 induces expression of genes associated with inflammation [58]. In this study, PLG inhibited the phosphorylation of MAPKs (ERK, P38) in DNCB-induced dorsal skin tissue, and the expression of ERK was confirmed to have an inhibitory effect when at a high concentration of 200 mg/ml. In addition, PLG inhibited the nuclear translocation of NF-κB in TNF-α/IFN-γ-stimulated HaCaT cells. These results indicated that the effect of PLG could be mediated through inhibition of NF-κB nuclear translocation, which is a target for AD treatment.

HaCaT cells have been widely used in skin biology research [59]. Keratinocytes provide an important window into the environment by forming the body’s skin barrier against environmental damage [38]. Activated keratinocytes produce various cytokines and chemokines to defend against inflammation, which are involved in the initiation and pathogenesis of inflammation [60, 61]. The MAPK/NF-κB signaling pathway also promotes AD through the production of pro-inflammatory cytokines and chemokines [62]. Thymus and Activation-regulated Chemokine (TARC)/CCL17, Macrophage-derived chemokine (MDC)/CCL22, and Regulated upon Activation, Normal T Cell Expressed and Presumably Secreted (RANTES)/CCL5 are inflammatory chemokines involved in Th2-dominated immune responses [63, 64]. It is expressed in keratinocytes stimulated by inflammatory cytokines to attract Th2 cells to infiltrate skin lesions [64]. Monocyte chemoattractant protein-1 (MCP-1/CCL2) is an influential chemotactic factor for monocytes belonging to the CC family chemokines [65]. MCP-1 is also known to regulate the migration and invasion of memory T cells and natural killer cells, and has been observed in study patients with chronic inflammatory skin disease [65, 66]. Macrophage inflammatory protein-3 alpha (MIP-3α)/CCL20 is a C–C chemokine expressed by keratinocytes and exhibits antibacterial activity against bacteria and fungi [67]. It is also an important chemokine for innate and acquired immune responses by migrating Langerhans cells into the skin [67]. In this study, the expression of RANTES, TARC, MDC, MCP-1, and MIP-3α in keratinocytes was inhibited by PLG. These results indicate that PLG can reduce the production of inflammatory chemokines by inhibiting the expression of MAPK/NF-κB pathways.

To identify and quantity the active compounds in PLG extract, we performed HPLC analysis. As a result, we found that epigoitrin and adenosine were present in PLG extract and confirmed the quantities using a standard curve. Based on previous discussion, epigoitrin was a marker compound in PLG according to the 2020 edition of the Chinese pharmacopoeia [68]. In addition, it has been known to be effective against viral infections caused by changes in the immune system in both in vitro and in vivo experiments [69]. Adenosine is widely known as a primary nucleoside in PLG, producing a variety of important biological effects. Adenosine has been known to have paroxysmal supraventricular tachycardia, phagocytosis, and anti-inflammatory effects [70, 71]. In particular, it has been known that adenosine receptors exist in cells and organs and play a powerful role in regulating inflammation by inhibiting inflammatory cell functions [71]. Therefore, epicoitrin and adenosine of PLG have been widely used as therapeutic agents for many diseases due to their various actions, but additional experiments were conducted to confirm whether they have anti-atopic dermatitis pharmacological activity. In this study, the effects of adenosine and epigoitrin on the production of proinflammatory chemokines induced by TNF-α/IFN-γ in HaCaT cells were investigated. As a result, adenosine decreased RANTES, TARC and MCP-1, and epigoitrin decreased TACR, MDC and MCP-1. However, epigoitrin increases the production of RANTES, requiring further experiments. Taken together, it has been demonstrated that PLG exerts anti-atopic dermatitis effects due to the activity of standard compounds adenosine and epigoitrin.

## Conclusion

We observed that PLG ameliorated AD-related symptoms and decreased activation of inflammatory mediators in DNCB-induced mouse models and TNF-α/IFN-γ-induced HaCaT cells. Consequently, this study demonstrated that PLG alleviated the symptoms of AD and reduced the Th2 immune response. Therefore, PLG is expected to be a safe and effective pharmacological agent for the treatment of AD.

## Supplementary Information

**Additional file 1: Table S1.** HPLC conditions for analysis standard compound and PLG extract.

## Data Availability

Not applicable.

## Abbreviations

AD: Atopic dermatitis
PLC: *Isatis tinctoria L*
DNCB: 2,4-Dinitrochlorobenzene
IgE: Immunoglobulin E
TNF-α: Tumor necrosis factor-α
MARK: Mitogen-activated protein kinase
p-ERK: Phosphorylation-ERK
p-P38: Phosphorylation-P38
NF-κB: Nuclear factor-κB
CMC: Carboxymethyl cellulose
DEX: Dexamethasone
H&E: Hematoxylin and eosin
TB: Toluidine blue
TARC/CCL17: Thymus and activation-regulated chemokine
MDC/CCL22: Macrophage-derived chemokine
RANTES/CCL5: Regulated upon activation normal T cell expressed and presumably secreted
MCP-1/CCL2: Monocyte chemoattractant protein-1
MIP-3α/CCL20: Macrophage inflammatory protein-3 alpha
