# Supplementary material for: Inhibitory effect of Isatis tinctoria L. water extract on DNCB-induced atopic dermatitis in BALB/c mice and HaCaT cells
Source: Chin Med. 2022 Jun 8;17:66. doi: 10.1186/s13020-022-00624-5 (PMC9175348; doi:10.1186/s13020-022-00624-5)
Supplement: Supplementary file 1 — Additional file 1: Table S1. HPLC conditions for analysis standard compound and PLG extract. [file 13020_2022_624_MOESM1_ESM.docx]

Table S1. HPLC conditions for analysis standard compound and PLG extract.

| **HPLC Conditions** | | | |
| --- | --- | --- | --- |
| **Detector** | 245nm | | |
| **Column** | X-bidge C18 Column (250 mm × 4.6 mm, 5 μm) | | |
| **Column Temperature** | 25℃ | | |
| **Injection Volume** | 10μL | | |
| **Flow rate** | 1.0mL / min | | |
| **Mobile phase** | Time (min) | A | B |
| **A: Water**  **B : Methanol** | 0.0 | 97 | 3 |
|  | 12.0 | 90 | 10 |
|  | 17.0 | 80 | 20 |
|  | 25.0 | 80 | 20 |
|  | 35.0 | 70 | 30 |
|  | 40.0 | 60 | 40 |
|  | 50.0 | 0 | 100 |
